# Theory and Treatment of Diabetes

**Published:** 1914-02

**Authors:** William Edward Fitch

**Affiliations:** New York


For Texas Medical Journal.

### Theory and Treatment of Diabetes.

BY WILLIAM EDWARD FITCH, M. D., NEW YORK.

"Laboratory work upon diabetes during recent years, and a more careful study of its metabolic disturbance, have taught us many new facts, particularly from the dietotherapeutic viewpoint which had been thought to be the true one, but in the light of later research has been materially modified. Therefore, what to many may pass as the newest standpoint is abandoned by others in favor of a still newer one." This is an introductory paragraph of a valuable contribution on the above subject read before the Medical Association of the greater city of New York, October meeting, appearing in the *New York Medical Journal* for the issue of December 13, 1913, by William Edward Fitch, M. D., of 355 West One Hundred and Forty-fifth Street, New York City, who, referring to the "Theory of Diabetes," says:

"There is no subject in medicine upon which have been contributed so much thought, time, and energy, from an experimental standpoint, as diabetes, and yet no subject as to the true pathology and etiology of which we possess proportionately less accurate information." For the sake of convenience, the author refers to three types of diabetes: 1. Cases due to lesions of the fourth ventricle. 2. Cases due to metabolic disturbances of the liver and alimentary canal. 3. Cases due to pancreatic disturbances (atrophy of the islands of Langerhans).

While the foregoing classification, for the sake of convenience, is referred to as types of diabetes, the author does not wish it inferred that he believes the diseased process is confined to any one organ, for no organ works for itself alone. It depends on processes taking place in other organs together with the products of its own activity, which influence the functions of other organs. These products may be *ferments* stimulating decomposition in the blood, or *hormones* regulating activity of the cells.

The author holds that metabolism is the all important factor in the maintenance of life and health. Heat and energy, he affirms, become absolutely necessary in order to convert the nitrogenous or protein elements into proteids and thence into tissue and fat. In order to get this heat and energy, the body burns carbon and thereby gets the heat, while the energy is supplied as the direct result of combustion by water contained in the starch elements.

In the process of digestion starches are converted first of all into alcohol before work on the rehabilitation of the organism can begin. We know that any food becomes an irritant when taken in excess above a legitimate demand; therefore, carbohydrates containing starch (untreated) given in great quantities to a diabetic will produce an irritating effect upon the sugar-producing organs; as a result the patient will suffer from glycosuria.

Why does a diabetic void sugar in his urine? asks the author. Then he answers, because of faulty carbohydrate metabolism, the patient is not able to convert the sugar into alcohol in his system. The reason he cannot is at present unknown to physicians and pathologists. This much is certain, however: If carbohydrates are withdrawn from his dietary, the patient will feed on himself until all the fat stored in his system has been consumed, and then he will die. While we know that alcohol has its abuses, yet we cannot live and have our being without alcohol in our system. We understand that all starches are first converted into alcohol before they can yield their energy to the system, and a certain amount of alcohol is necessary for the conversion of nitrogenous food into tissue-building material to sustain the human economy.

The treatment of diabetes is far from ideal, affirms the author, being a malady subject to varied complications, it necessarily presents a difficult problem in treatment. We recognize that it is primarily a disturbance of nutrition in which the function of utilizing carbohydrate foods is more or less completely impaired. No disease, says the author, with which he is conversant necessitates such a careful balancing of all the details of each individual case, the metabolic powers of the patient, the functional condition of the liver, the condition of the alimentary tract, the state of his nervous system and of his environment, and the functional capacity of his pancreas, must be severely and collectively considered in forming a conclusion as to the advice that should be given him for his daily conduct in the future.

The newest researches have taught us, says the author, that the manner in which carbohydrates are allowed is of the greatest importance. The human body obtains its energy and nutrition from three types of foodstuffs—proteins, fats, and carbohydrates.

For general purposes work, warmth, and nutrition, all three serve well; the muscular tissues are, however, most specific in their requirements. They have a predilection for sugar as their foodstuffs and immediate source of energy. It is necessary, therefore, that sugar should always be available for this purpose. The liver with its sugar factories—the liver cells—fulfills this function.

The author discusses the various carbohydrate cures which have been recommended in diabetes. The oatmeal cure by Von Noorden, the rice cure by Von During, the potato cure by Mosse and others; but his experience is that none of them can be relied upon.

The author then refers to a carbohydrate food which has given pleasing results in the dietetic treatment of this malady. This particular food is made from whole grain wheat and barley cereals. The grain is ground on the old-fashioned buhr millstone, and the finished flour contains all of the starch and cereal salts that nature grew into the grain in the field. After being ground, the whole-wheat flour is subjected to a series of special processes brought about by the application of a certain degree of heat for a specified period of time, which causes certain changes in the starch atoms. This special treatment by the application of heat produces changes in the envelope of the starch molecules which facilitate digestion and assimilation. The author believes in allowing the diabetic carbohydrate, together with nitrogenous food, sufficient to build up and maintain the human economy; but starch and nitrogen *per se* mean nothing toward sustenance until the animal economy has converted these elements into tissue-building material. Among the many advantages of starch-treated foods is the fact that they indirectly facilitate the absorption of other foods, especially carbohydrates of every kind, on account of the apparent inducement they seem to hold out to the enzymes of the intestinal tract, to assume their full amylolytic function with promptness and thoroughness. The author has traced a great number of cases of various types of diabetes by prescribing these starch-treated foods to the exclusion of all other starch foods, and has been very much gratified to see the percentage of sugar in the urine gradually decrease and finally disappear.

---

"Bier uses local anesthesia if possible with novocain, one-half per cent. He does not often use spinal anesthesia, being so well satisfied with the local. I witnessed the removal of a kidney under local anesthesia."—W. J. Mayo, in *Journal Lancet,* August 15, 1912.

---

In preliminary ligation for exophthalmic goitre, by C. A. McWilliams, "the right superior thyroid vessels were tied on August 5, 1908, using local anesthesia with a one-eighth per cent solution of novocain, combined with adrenalin, and without the preliminary use of morphine. No symptoms resulted from this procedure, nor was there any improvement in her condition, as was to be expected. Accordingly, ten days later, the left superior thyroid vessels were similarly ligated, under the same local anesthetic. She immediately began to improve, mentally and physically, and in seven weeks had gained 16 pounds."—Proceedings of New York Surgical Society, May 8, 1912.

